# Achieving 90–90–90 in paediatric HIV: adolescence as the touchstone for transition success

**DOI:** 10.7448/IAS.18.7.20257

**Published:** 2015-12-02

**Authors:** Sonia Lee, Rohan Hazra

**Affiliations:** Eunice Kennedy Shriver National Institute on Child Health and Human Development, Maternal and Pediatric Infectious Disease Branch, Bethesda, MD, USA

**Keywords:** children, adolescents, HIV, transition, guidelines

## Abstract

**Introduction:**

The number of children less than 15 years estimated to be living with HIV globally approximated 3.2 million in 2013. Young people aged 15 to 24 years living with HIV approximated 4 million. The survival of these children and adolescents into adulthood poses new and urgent challenges of transition from the paediatric to adolescent to adult healthcare settings due to emerging developmental, psychosocial and comorbid issues. In order to achieve treatment targets of 90–90–90 across the continuum of care for paediatric HIV by 2020, focused efforts on the implementation of appropriate healthcare transition plans across the lifespan, with a focus on adolescence, should be prioritized.

**Discussion:**

Published data or empirical evidence examining implementation of transition models and association with clinical outcomes are limited. While some guidelines do exist that offer recommendations about how to promote seamless transitions, very few data are available to assess the adequacy of these guidelines and whether they are effectively adhered to in clinical care settings globally. Furthermore, paediatric and adolescent HIV infection, either acquired perinatally or behaviourally, is set apart from other chronic illnesses as a highly stigmatizing disease that disproportionately affects poor, minority and often marginalized populations. Focused efforts on adolescence as the touchstone for transition practices and policies need to be implemented.

**Conclusions:**

Optimal healthcare for these vulnerable populations, particularly in resource-limited settings, will require HIV-specific transitional care services and programmes that are coordinated, collaborative, integrated and, importantly, evidence-based.

## Introduction

Survival of perinatally HIV-infected (PHIV) children into youth, and continued survival of PHIV youth and behaviourally HIV-infected youth into adulthood, poses new challenges of transitions from the paediatric and adolescent healthcare setting to the adult healthcare setting. These transitions across the early lifespan encompass a dynamic developmental stage, namely adolescence, in which youth are establishing their identity and autonomy, mastering abstract thought, negotiating independent decision-making, managing educational and employment challenges and having intimate relationships [1]. For these HIV-positive youth, this complex developmental stage also occurs in the context of their HIV infection and often economic, social and familial stress. Additional issues such as disclosure of HIV illness and stigma also need to be navigated. Therefore, optimal healthcare for these children and youth must consider healthcare transition as a continuum and not separate, discrete moves from paediatric to adolescent to adult clinic settings. Such consideration will include the development of a formal plan among the child, family and medical providers with a focus on the adolescence transition period.

It is estimated that in the United States over 90% of children with chronic conditions will survive beyond their twenties [2]. Due to medical advances and the increased availability of antiretroviral medications, the overall number of children and youth with HIV surviving into adulthood is increasing. The reduction in AIDS mortalities in infants in South Africa has shifted the burden of paediatric HIV to older children with a growing proportion of AIDS deaths in older children [3]. Globally, improved care and treatment options have increased the lifespan of people living with HIV, with rapid decreases in AIDS-related deaths between 2001 and 2013 for all age groups, with one notable exception – adolescents (aged 10 to 19 years) [4]. In response, the 90–90–90 targets, developed by UNAIDS and partners for all people living with HIV, have been adopted: by 2020, 90% of all people living with HIV will know their HIV status; 90% of all people with diagnosed HIV infection will receive sustained antiretroviral therapy (ART); 90% of all people receiving ART will have viral suppression [5]. The 90–90–90 treatment goals must include children and adolescents. They can only be achieved if appropriate transition plans are methodically incorporated into the healthcare of children and youth with HIV.

Over the past 20 years the Society for Adolescent Medicine [6,7], the US Department of Health and Human Services [8], the American Academy of Pediatrics [9] and others [10] have made efforts to promulgate the critical need to study and evaluate transition processes. Despite these efforts, transitional care services and programmes that are coordinated, collaborative, integrated and, most importantly, evidence-based are lacking [11]. Indeed, the immense burden of transitioning patients across various healthcare clinics needs to be removed from medical care providers and even the patients themselves. Instead, the HIV community of researchers, policy-makers and broader healthcare service programme advocates need to take the lead. The effectiveness of integrating implementation science research efforts with current policies, guidelines and recommendations for effective transitioning needs to be assessed. The number of children with HIV ageing into adolescents, in addition to the increasing number of new HIV infections in adolescents, is developing into an enormous burden on current healthcare services and programmes worldwide [12,13]. Indeed, disparities and trends in AIDS mortality among adolescents with HIV in low- and middle-income countries call for immediate action [14]. It is imperative to conduct research studies to evaluate not only the transition process, but also the outcomes of transition, including the 90–90–90 targets, in order to implement findings and determine the optimal healthcare approach to manage this burgeoning change in the landscape of paediatric HIV.

## Discussion

### Emerging health challenges

Paediatric HIV poses a specialized challenge from other chronic illnesses in that treatment, and thus transition, plans should be mindful of emerging health challenges such as cardiovascular disease risk and bone health [15–18]. In addition, biomedical comorbidities represent a particularly important and relevant concern to manage over the course of HIV infection and thus to address in any transition plan concerns psychiatric illnesses. Children and youth with HIV are at increased risk for psychiatric hospitalizations, compared with the general paediatric population [19]. A systematic review of patients with HIV infection and psychiatric diagnoses and symptoms also found an increased risk of psychiatric illness among HIV-positive individuals than among the general population [20]. With improved survival among children and youth with HIV, the manifestations of neuropsychiatric complications during HIV disease, including HIV-associated neurocognitive disorders, and the use of psychotropic medications in conjunction with antiretroviral treatments, are additional outcomes to screen for and manage in an effective and adaptable treatment and transition plan.

### Emphasis on adolescent-friendly services

Adolescents who acquired HIV infection horizontally through risk behaviours (such as consensual or non-consensual sex and injection drug use) are a distinct population from adolescents with perinatally acquired HIV. However, the many challenges of moving to adult healthcare, including disclosure of HIV status to others and emerging independence in managing a chronic illness, will need to be integrated into a purposeful and planned transition. Adolescence poses a developmental and high-risk period where physical, emotional and social stressors may impact how youth cope with their HIV and manage their treatment. Other factors for adolescents to tackle during this time include stigma, feelings of loss and death and lack of social support [21]. Overall, adolescents are significantly underserved by HIV services and thus have poor access to treatment options and lower adherence to ART and medical appointments than adults [22]. Indeed, the HIV cascade of care from HIV diagnosis to viral suppression among adolescents and young adults indicates larger declines at all steps than older adults with HIV [23]. Therefore, it is important for transition plans to bridge these gaps by adapting and tailoring HIV services to retain adolescents in care, improve adherence to ART and manage the developmental issues in parallel to their chronic illness. A comprehensive approach to transition to assist healthcare providers as well as children, adolescents and their families will ensure a seamless and successful integration into a new, adult care setting. Complexities inherent with successful transition outcomes and the ultimate goal of viral suppression become most apparent for adolescents with HIV, including awareness that many paediatric, adolescent (if available) and adult HIV care models have fundamental differences [24]. For example, most adolescent HIV clinics are multidisciplinary and cater to more than HIV treatment with an integrated approach to address sexual/reproductive health, mental health-related issues and adherence counselling. On the other hand, many adult clinics may prefer to refer the young adults to separate subspecialty care settings. This change in medical care provision practice places additional burdens on the newly transitioned young adult to take ownership of and integrate their care, practice autonomy and independent decision-making and provide informed consent for new procedures and doctors. The pressure for young adults to independently navigate a fractionated healthcare system may potentially increase the lack of treatment engagement altogether [25]. As such, transition plans need to fill in the treatment gaps along the continuum of care trajectory by specifically targeting and focusing on adolescence by integrating “adolescent-friendly” healthcare services into a comprehensive care model [26]. This step is particularly important for those practices that directly transition patients from a paediatric care setting to an adult care facility, without separate consideration for adolescent-specific services. To date, too much focus for transition success has been placed on establishing guidelines for medical providers and for the patients themselves. Instead, the focus needs to shift on implementation science research efforts as the context of HIV care varies incredibly due to resources, healthcare provision and policies and geographical settings.

### Striving for healthy outcomes

In addition to encompassing the complex developmental, psychosocial and medical issues facing children and adolescents with HIV as they transition to adulthood, a comprehensive care transition programme should be guided by further research on the predictors of healthy outcomes. Especially with respect to strategies for achieving the 90–90–90 treatment targets, more needs to be known regarding how these vulnerable HIV populations fare with respect to their health, physical and mental, in adulthood. Unfortunately, preliminary findings point to the transitions from paediatric to adult care as yet another risk factor for poor clinical outcomes [27]. A study conducted in England highlights poor consequences for young people with perinatally acquired HIV infections following their transfer to adult care [28]. In this study, the estimated minimum mortality rate for those PHIV patients aged greater than or equal to 21 years in adult care was nearly five times greater than those PHIV patients aged 13 to 15 years in paediatric care. Better methods for continued surveillance of PHIV populations to reliably and systematically track outcomes will greatly inform the current guidelines and recommendations for transition to adult care settings [29–31]. In addition, published data and/or empirical evidence examining implementation of transition models and association with clinical outcomes are critical. Despite previous recommendations for this type of research, published data on healthcare transition outcomes for HIV populations are limited and, thus, evidence-based transition recommendations are currently lacking.

A crucial research area to improve transition models encompasses the voices of youth actively going through the transition process as well as young adults who have just experienced the transition process. Qualitative interview responses, demographic information and physical health status outcomes were collected from a study to explore the experiences of 42 recently transitioned youth (mean age 22 years) [32]. Unfortunately, the health outcomes (measured by CD4 counts) trended in the downward direction with 45% reporting a more difficult transition process than anticipated. Several barriers to successful transition were cited: economic, logistical, lack of communication between paediatric and adult care providers, lag in developmental readiness, difficult access to psychosocial services and even reluctance by paediatric providers to transition their patients who had been under their care for most of their lives. Separate qualitative studies conducted with small samples of transitioned young adults with behaviourally acquired HIV (ages 24 to 29 years) emphasized how they felt unprepared for transition, resulting in increased anxiety about expectations for their future [33–35]. Important concepts of readiness [36–38] and stigma [20] should also be continually assessed prior to and during the transition process. Additional needs for successful transition plans should also include an assessment of the young adult's educational aspirations, with appropriate vocational and life skills training. As the prognosis for children and adolescents with HIV has changed, paediatric care providers will need to work with adult care providers to better prepare their patients for independent and healthy living.

### Integrated management of illness

With HIV as the second leading cause of death among adolescents in 2012 [39], international efforts to improve surveillance of the global adolescent health epidemiology are needed, as are efforts to define public health priorities for adolescents. Indeed, as many adolescent health problems start from childhood and continue across the life course into early adulthood, transition programmes need to emphasize the impact of developmental processes on the care continuum. Previously mentioned guidelines and recommendations for transition have emanated from the United States and tend to focus on medical providers. Instead, their applicability to international settings most affected by HIV needs to be carefully evaluated and the contexts under which transition occurs (or does not successfully occur) need to be considered. An integrated management of adult, adolescent and childhood illness [40] has been developed to support a public-health approach in resource-limited, developing countries, where the HIV pandemic is more severe. This poses a different model from the tailored approaches recommended in the United States, where resources to modify healthcare services are more readily available. Since 2001, the WHO has promoted a conceptual shift from an individual-based to a population-based approach to ensure access to ART treatment, outside the specialized management approach of high-resource settings [41]. For example, the Thai HIV programme provides free, comprehensive HIV treatment for children, delivered by their Ministry of Public Health through a holistic model, including clinical and psychosocial care; this model is evolving to encompass the entire care continuum from infection with HIV through the transition to adult services [42]. Individual-based approaches to HIV care pose challenges, including the need for numerous providers with separate, specialized expertise. One unique and important aspect of this public health approach to HIV is through the integration of volunteer “co-providers” at clinics and in the community as support systems for patients across developmental stages. Further evaluations and evidence for the success of a public-health approach to scale up HIV services and facilitate access to ART are becoming more widespread. Parallel efforts should also be implemented globally to address the evolving needs of those living with HIV and to ensure the smooth transition of children to adolescents to adults with simple, standardized, decentralized and equitable healthcare.

## Conclusions

While some guidelines do exist to offer recommendations about how to manage the transitions across different healthcare settings, very few data are available to assess the adequacy of these guidelines and whether they are routinely implemented in clinical care settings, both domestically and globally. In order to attain the 2020 targets of 90% of all people living with HIV aware of their HIV status, 90% of all people diagnosed with HIV on sustained ART and 90% of all people living with HIV achieving viral suppression, HIV research priorities need to emphasize empirical investigations of healthcare setting transition plans (see Table 1).

**Table 1:** Future priorities for transition research and practice

•	Evidence-based transition models specifically for HIV populations, with a focus on adolescent development care and needs
•	Longitudinal studies, including registries and/or cohorts of children and youth with HIV to effectively track and monitor physical and mental health outcomes across the lifespan
•	Adolescent-friendly HIV healthcare services with comprehensive care to incorporate emerging co-morbidities
•	Broader health system reform and policies to approach HIV care from a dynamic, developmental perspective and to consider social and contextual factors such as stigma, resources and access

Focused transition efforts within the field of HIV have the potential to address the broader issues of chronic illness during adolescence, especially in low- and middle-income countries, where adolescent health services are quite limited. Optimally, with adequate preparation, children and youth with HIV will have appropriately transitioned to adult care upon successful implementation of a well-prepared plan, developed over the entire life course of their HIV illness. They will feel clinically and psychosocially able to manage their HIV illness, seek and attend appropriate medical appointments and will become goal-oriented toward healthy and long-term living. Targeting the developmental period of adolescence for vulnerable populations of children with HIV and focusing our efforts of improving HIV care services [43], in conjunction with initiating an overarching umbrella of broader health system reform and policies [17], will facilitate transitioning children to adolescents, and adolescents to adults.
